# Feasibility and utility of telemedical support through a paramedic-led primary response service to aid emergency services in northwestern Germany

**DOI:** 10.1186/s12913-025-13748-9

**Published:** 2025-12-01

**Authors:** Daniel Overheu, Simon T. Schäfer, Bastian Rosner, Tobias Warnecke, Nils Jacobsen

**Affiliations:** 1 Carl von Ossietzky Universität Oldenburg School VI - School of Medicine and Health Sciences, Ammerländer Heerstraße 114–118, 26129 Oldenburg, Germany; 2 Clinic for Anesthesiology, Emergency Medicine and Pain Therapy, Evangelisches Krankenhaus Oldenburg (EVK), Steinweg 13 – 17, 26122 Oldenburg, Germany; 3 University Clinic for Anesthesiology, Intensive Care Medicine, Emergency Medicine, Pain Therapy, Klinikum Oldenburg AöR, Rahel-Straus-Straße 10, 26133 Oldenburg, Germany; 4 Department of Intensive Care and Emergency Medicine, Klinikum Bremen-Mitte (KBM), Sankt-Jürgen-Straße 1, 28205 Bremen, Germany; 5 Institute for Rescue and Emergency Medicine, Universitätsklinikum Schleswig-Holstein (UKSH), Holzkoppelweg 8 – 12, 24118 Kiel, Germany

**Keywords:** Community paramedicine, Emergency service, Rural areas, Medical on-call service, Association of statutory health insurance physicians

## Abstract

**Background:**

In Germany, the regional Associations of Statutory Health Insurance are responsible for providing an out-of-hours on-call service for family medicine. With a shortage of resident doctors in rural areas, maintaining this service becomes more challenging. This raises the question whether the combination of a physician-run telemedical support with a primary response service led by paramedics is feasible, and in which way the telemedical support affects decision-making on scene.

**Methods:**

We implemented Germany’s first Standard Operating Procedure (SOP)-based on-call service for paramedics on site with telemedical backup. Between March and June 2018, differentiated SOPs and a special medical backpack were established. Participating paramedics and telemedicine doctors were trained in SOPs and the use of telemedical software. Descriptive statistics were used to analyse data collected during the study period from July 2018 until December 2021.

**Results:**

Overall, 17% of all cases required telemedical support, primarily for diagnostic confirmation of musculoskeletal disorders, unclear symptom constellations, and diseases of the cardiac circulatory system. In 56% of all telemedicine-assisted cases paramedics on scene altered their originally planned course of action following telemedical support.

**Conclusion:**

This study demonstrates that telemedical assistance is a feasible alternative to the long-established doctor-run on-call system which is currently implemented throughout Germany. In the majority of cases, the telemedical input had a significant impact on the decision-making process of the responsible paramedic on scene. Limitations arise through limited information on some variables driving that decision-making process. Furthermore, the number of telemedical personnel required to facilitate the provision of telemedical assistance across an entire national healthcare system remains unclear.

**Trial registration:**

German register for clinical trials DRKS00024037, registered March 18th 2022 (BfArM - Deutsches Register Klinischer Studien (DRKS).

## Background

Due to its diversity, the German healthcare system is unique in its emergency outpatient services. In Germany, emergency care is delivered on three distinct levels on a 24/7 basis. The first level consists of an ambulance rescue service provided by emergency physicians and paramedics, the second of emergency departments at hospitals and the third is a general practitioners (GP) on-call service provided by the regional Associations of Statutory Health Insurance Physicians. The latter provides primary care by doctors whenever the local GPs’ offices are closed. With the ever-growing problem of an aging GP workforce in Germany [1] it is increasingly difficult to find successors for GPs who are willing to settle down in rural areas and thus, can provide primary care for the rural population. Furthermore, the GP-on-call services, which need to be provided for up to 24 h, and the work carried out at the GP’s own practice are often performed by the same individual. Consequently, there is an increasing shortage of resident doctors available for this on-call service in rural areas.

Also, patients in Germany can freely choose which type of medical service they require (i.e. ambulance services, emergency departments at the hospital, or the GP on-call service). A common issue is a lack of public understanding on when to use which type of emergency service [2] which leads to misutilisation. This problem is further enhanced by missing integrations between GP-run out-of-hours services and the emergency medical services dispatch centre. Thus, patients unnecessarily use paramedic service or emergency departments instead of contacting the GP on-call service, which is implemented to provide primary family care [3]. This leads to overcrowding in A&E and inefficient use of non-emergency on-call services [4, 5].

In light of these challenges, the question arises whether alternative organisational models need to be considered. This study evaluates the feasibility of a paramedic-led primary response service supported by telemedical consultation. The advantage would be, that the telemedical support can cover large areas mitigating the lack of available GPs.

In our specific project the city of Delmenhorst as well as the neighbouring municipalities (overall about 120,000 inhabitants [6]) proved ideal due to persistent staffing difficulties in the GP out-of-hours service. The responsible local bureau of the regional Association of Statutory Health Insurance Physicians (KVN Oldenburg), together with the University Clinic for Anesthesiology of Klinikum Oldenburg AöR (KOL) and the St. Johns Ambulance Germany (JUH) started a funded project to staff this local area with paramedics and telemedical assistance.

Our study analyses the effect of telemedical support on the clinical decision-making process of paramedics working in collaboration with this GP-on-call service over a follow-up period of 42 months. To the best of our knowledge, our study represents the first investigation of this innovative approach in Germany.

## Methods

The local ethics committee of the Carl von Ossietzky University of Oldenburg confirmed that no formal approval of the study was needed due to the anonymous retrospective design (AZ 2021-012). The study was registered at the German register for clinical trials under the number DRKS00024037.

The three partners KVN, JUH, and KOL conducted four project meetings where recently published data and Standard Operating Procedures (SOPs) were examined and tested for their feasibility when applied to the GP on-call-service. Existing general and emergency medical SOPs for paramedics [7, 8] were checked, evaluated, amended, and finally converted into SOPs tailored to the work in the on-call service. In total, 10 SOPs for the following topics were provided: acute diarrhea, agitation, analgesia, handling urinary catheters, headache, airway infection, soft tissue injury, sore throat, urinary tract infection, and wound care.

To ensure the secure implementation and utilisation of SOPs and telemedical support provided, it was mandatory for all healthcare professionals assigned to this project to be qualified as state-certified nurses, emergency paramedics, or geriatric nurses.

**Fig. 1:**
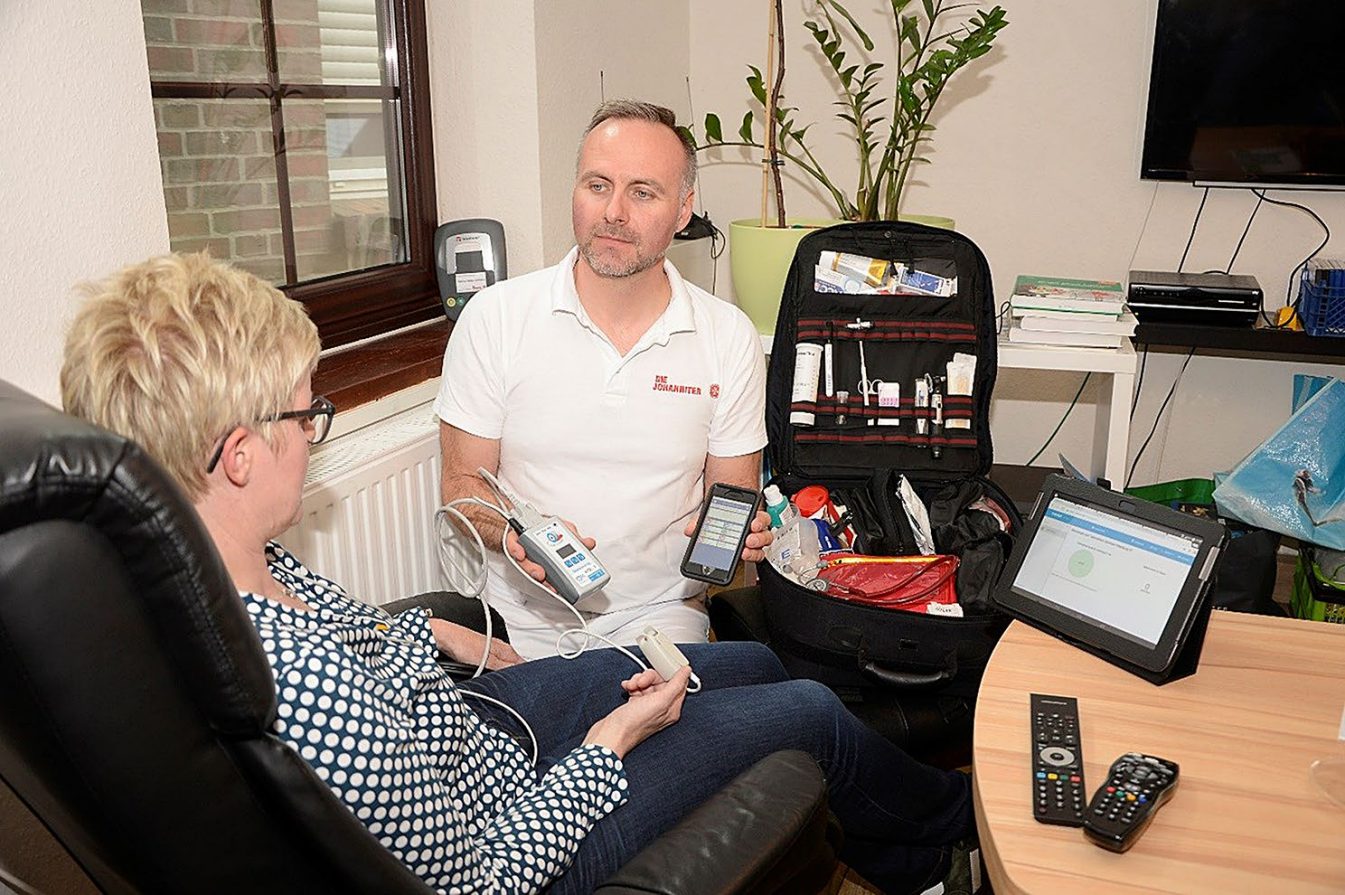
Healthcare professional on site. (Source: JUH, Stefan Greiber)

Further on, the project partners agreed on a material concept and the equipment of a specified on-call emergency backpack. In addition to the equipment specified in the SOPs, the backpack included the necessary technical components to receive telemedical support on site at all times (see Fig. 1). For streaming vital signs, the following equipment was procured: 12-lead ECG, pulse oximetry and calculated blood pressure via pulse transit time method [9], and integrated in a DynaVision X device (DynaVision X, Techmedic International BV, The Netherlands). Also, a standard tablet (iPad, Apple Inc., California, USA) with secure audio/video streaming software (Corpuls Mission, GS Stemple GmbH, Germany) was provided for all participants. All healthcare professionals were introduced to and trained with the technology and software. Before commencing field work, all components needed for storage and transfer of personal health data were checked by the data protection officer of the KOL to ensure compliance with the applicable data protection regulations.

Telemedical expertise was ensured by telemedical support services of the University Clinic for Anaesthesiology, Intensive Care Medicine, Emergency Medicine and Pain Therapy at Klinikum Oldenburg AöR. The specialists in anaesthesiology and intensive care medicine were extensively trained in project SOPs, the accompanying software for audio/video streaming, and data reception from the mobile telemetry devices. If necessary, experts from other specialist departments (e.g. dermatology, cardiology, ENT, etc.) at Klinikum Oldenburg could be consulted on relevant issues.

Joint system tests were carried out together with participating healthcare professionals at various points in the area of ​​operation to test whether GSM (Global System for Mobile Communications) connection can be assured at all times. The project started on July 1st, 2018 and the on-call service was implemented in a two-step fashion. First, GP-on-call was only run from Friday 7 p.m. till Monday 7 a.m for the communities Delmenhorst and Lemwerder. Starting April 1st, 2020 the service was extended to out-of-office hours from Monday till Thursday, and three additional communities (Wildeshausen, Dötlingen and Grossenkneten) were included.

As there is no integration between the dispatch center for emergency medical services (when calling 112) and the dispatch centre for GP-on-call-services (when calling 116117), patients are free to choose which service to contact first. Dispatchers at 112 may then advise patients to call 116,117 if they assess a case as low acuity. Due to this system structure, only patients who called 116,117 and were located in the area of Delmenhorst and the surrounding communities received paramedic-led GP care during this project. In all other regions, a GP on call had to see the patients in person when dispatched via 116,117. Until 2020, it was the decision of the dispatchers which patient had to be consulted in person. In 2020, the software tool SMED (https://www.zi.de/themen/medizin/smed/uebersicht*)* was introduced and is now providing standardised recommendations on which patients need in-person assessment by a GP.

Each case over the 42-month project period (July 1st, 2018 to December 31st, 2021) was recorded in a report by the healthcare professionals on scene. Since January 1st, 2019, participants not only recorded medical data but also evaluated the effects, circumstances, and consequences of all assignments (see Table 1). In addition to tactical deployment and patient data, healthcare professionals also documented suspected diagnosis and incident characteristics using the SAMPLER scheme [10]. Vital signs obtained as part of the patient examination (respiratory rate, oxygen saturation, heart rate, blood pressure, blood sugar, temperature) were listed and monitoring methods used were recorded via checkboxes. Additionally, not only the date but also the operation length was documented including time of alarm, arrival time on site, and end of operation.

**Table 1:** Suspected diagnosis based on ICD-10 main groups

ICD-10 main group:	Total cases	Use of telemedical support			Usefulness of telemedicine		
		Tele-med. used	Valid answers	%	Coded as “helped accompl-ish mission”	Valid answers	%
J00-J99 Diseases of the respiratory system	302	18	266	6.77	3	6	50.00
M00-M99 Diseases of the musculoskeletal system and connective tissue	241	31	212	14.62	14	20	70.00
F00-F99 Mental and behavioural disorders	173	35	154	22.73	13	19	68.42
I00-I99 Diseases of the circulatory system	161	44	143	30.77	16	22	72.73
S00-T98 Injury, poisoning and certain other consequences of external causes	160	15	137	10.95	4	6	66.67
K00-K93 Diseases of the digestive system	156	19	139	13.67	7	7	100.00
N00-N99 Diseases of the genitourinary system	122	15	107	14.02	3	4	75.00
A00-B99 Certain infectious and parasitic diseases	112	20	103	19.42	9	12	75.00
Z00-Z99 Factors influencing health status and contact with health services	79	7	54	12.96	6	6	100.00
E00-E90 Endocrine, nutritional and metabolic diseases	60	18	57	31.58	7	8	87.50
G00-G99 Diseases of the nervous system	47	11	43	25.58	7	8	87.50
L00-L99 Diseases of the skin and subcutaneous tissue	38	12	33	36.36	6	8	75.00
H60-H95 Diseases of the ear and mastoid process	14	2	13	15.38	1	1	100.00
U00-U99 Codes for special purposes	14	1	14	7.14	0	1	0.00
H00-H59 Diseases of the eye and adnexa	12	1	11	9.09	1	1	100.00
O00-O99 Pregnancy, childbirth and the puerperium	8	1	7	14.29	1	1	100.00
C00-D48 Neoplasms	6	2	5	40.00	0	1	0.00
D50-D90 Diseases of the blood and blood-forming organs and certain disorders involving the immune mechanism	2	1	1	100.00	1	1	100.00
V01-Y84 External causes of morbidity and mortality	2	1	2	50.00	1	1	100.00
Q00-Q99 Congenital malformations, deformations and chromosomal abnormalities	1	1	1	100.00	0	0	0.00
P00-P96 Certain conditions originating in the perinatal period	0	0	0	0.00	0	0	0.00
R00-R99 Symptoms, signs and abnormal clinical and laboratory findings, not elsewhere classified	818	112	663	16.89	42	60	70.00
**Total**:	**2528**	**367**	**2165**	**16.95**	**142**	**193**	**73.58**

The objective of this study was to evaluate the impact of telemedical support on the visit itself and on subsequent treatment. Specifically, we examined whether telemedical support facilitated the completion of visits, whether alternative standard operating procedures might have been more appropriate, and whether telemedicine was perceived as helpful overall. Finally, we compared these telemedicine effects and suspected diagnoses (ICD-10 main groups [11]) to analyse in which cases telemedicine proved to be most effective. We calculated the proportion of missions coded as “helped accomplishing this mission” as follows:

Percentage helpful = 100 * (Missions coded as helped accomplishing this mission) / (Total number of missions with telemedical support, within the same diagnostic group, with ≥ 10 responses to the evaluation questions).

From January 1st, 2019 a questionnaire on details of the telemedical support was part of the on-scene documentation (no systematic assessment of the visits in 2018). Available questionnaire data were anonymised and transferred to a database and analysed using Microsoft Excel (Microsoft Excel 365, Microsoft Corp., California, USA). For a more detailed analysis, cases were split into groups based on suspected diagnoses according to the ICD 10 classification.

## Results

During the 42-month project period (July 1st, 2018 to December 31st, 2021) a total of 2,528 patient visits were documented by the paramedic team. From January 1st, 2019 onwards, a total of 2,165 surveys regarding the possible use of telemedicine were analysed. Telemedical support was given in 367 of these missions (16.95%). As not all these questionnaires provided answers to all following questions, the number of responses is indicated separately for each question.

Treatment on scene was affected by telemedical support. In 56.16% of telemedicine supported missions (114 missions) consulting a physician via telemedical support changed the course of management compared to the initially planned treatment of the medical specialist on site. In 43.84% of missions (89 missions) the initial plan was confirmed by the physician and thus the procedure remained unchanged.

Overall, the effect of telemedical support was rated as “helpful to accomplish the visit” in 73.58% of the documented visits with telemedical support (142 missions). The rating “different SOP would have helped during this visit instead of telemedicine” was chosen in 13.99% of all cases using telemedicine and the rating “no help at all for this mission” was used in 12.44% of missions. The specific reasons why telemedical support was not considered helpful in certain missions (technical issues, medical problem not solved via telemedicine, specialist for specific medical problem not available immediately, etc.) were not systematically recorded.

Documented suspected diagnoses according to the ICD-10 classification main groups are shown in Table 1. Cross-referencing ICD-10 main groups with the use of telemedical support showed a strong correlation between the total number of cases within a diagnostic group and the number of telemedicine-supported visits (Pearson’s correlation coefficient 0.945). In other words, if there were a lot of cases from one specific ICD-10 group, there tended to be more telemedicine visits as well. Despite this, there were some differences across the groups. Looking at all ICD-10 groups with at least 10 visits, L00-L99 (Diseases of the skin and subcutaneous tissue) had the highest percentage of telemedicine consultations (36.36%) while only 6.77% of all visits for J00-J99 (Diseases of the respiratory system) were accompanied by telemedicine.

Furthermore, we investigated which groups might most benefit from telemedical support.

Telemedicine had the biggest impact on cases with the following diagnoses:

1. Certain infectious and parasitic diseases (A00-B99): 75.00%,
2. Diseases of the circulatory system (I00-I99): 72.73%,
3. Symptomatic cause of consultation (R00-R99 Symptoms, signs and abnormal clinical and laboratory findings, not elsewhere classified) and diseases of the musculoskeletal system and connective tissue (M00-M99): both 70.00%.

In 37.55% of missions (80 missions) a recommendation was given to see a doctor in person during practice hours. The extent to which the patients adhered to this recommendation was not monitored as part of this study.

## Discussion

This study is the first to analyse the effects of telemedical support on healthcare professionals working within the GP-on-call service of the regional Association of Statutory Health Insurance Physicians in northwestern Germany. Among the available 2,165 case reports with completed questionnaires, 367 (16.95%) missions reported the use of telemedicine. In 73.58% of cases with telemedical support the healthcare professionals rated the support as “helpful to accomplish this mission”, and the patient could remain at home. Moreover, in 56.16% of all missions telemedical support led to a change in the originally planned management of the case. Analysing ICD-10 subgroups, telemedical support seemed to be most valuable when diagnoses of ICD groups A00-B99 (infectious/parasitic), I00-I99 (cardiocirculatory system) or M00-M99 (musculoskeletal) were present. Furthermore, these subgroups not only accounted for a significant proportion of telemedical interventions but also showed high ratings for perceived usefulness in accomplishing the mission. Further studies are needed to investigate why telemedical support was particularly beneficial in these specific diagnostic groups. Our study shows the feasibility of paramedic-led care in combination with a telemedical on-call service in Germany. Internationally, video and telephone support for medical decision-making is already very common and widely used. Abrashkin et al. could not show a significant benefit of a video-capable community paramedic system in the U.S. when comparing the emergency department admission rates [12]. However, their study did not analyse the efficacy and usefulness of telemedicine when combined with vital data transmission.

In our study, telemedicine was most effective when used for suspected diagnoses of ICD-10 subgroups concerning infectious/parasitic diseases, the cardiocirculatory system and the musculoskeletal system. This is a rational conclusion given that the transmission and discussion of ECG, blood pressure, and oxygen saturation combined with video streaming enabled the telemedicine doctor, and if necessary, a specialist, to rule out serious diagnoses. A sufficient audio/video connection in combination with livestreaming of vital signs is therefore an adequate way to present the situation on site. Thus, telemedicine doctors can make qualified decisions regarding the health of the patient in the field without being present themselves. Only in 12.44% of all missions using telemedicine, the support was regarded as “not helpful at all” so that the patient had to be seen by a doctor in person.

With an overall rate of 37.55% of patients being referred for GP follow-up treatment, the results of this study are comparable to other healthcare professional-based systems in Germany, such as the “Gemeindenotfallsanitäter” (a community emergency paramedic service that is currently evaluated [13]) or community paramedic systems in the USA [14]. Due to strict regulations, a community paramedic in Germany is still a relatively new approach and is therefore currently limited to deployment via emergency dispatch centres (112) in certain regions of Lower Saxony. In contrast, our study describes the dispatch of paramedic led-care following a call to the GP out-of-hours number (116117). Ultimately, both systems will likely encounter the same patient populations, depending on which emergency number the patient choses to call. This highlights the urgent need to combine both systems in an interoperable and integrative manner in the near future to ensure efficient use of resources.

For Germany, the approach of using healthcare professionals instead of family doctors or specialists to run the out-of-hospital on-call service is completely new. The nationwide implemented number (116117) to reach the GP-on-call service is fairly unknown in the general population throughout Germany [15], which often leads to overcrowding in emergency rooms and the disproportionate use of emergency services. Every member of the regional Association of Statutory Health Insurance Physicians of Lower Saxony is obliged to serve in this on-call service [16]. Moreover, the existing doctor-based medical emergency service frequently provides patient treatment without predetermined SOPs but based solely on the doctor’s existing knowledge. However, previous experience in emergency medicine is highly variable, as medical subspecialties other than family medicine are also required to perform this emergency service.

Our study shows that paramedic-led care in combination with a telemedical on-call service is helpful in most cases and can thus help to relieve the burden on the general GP on-call service as well as emergency rooms. Overcrowding of emergency rooms will remain a challenge in the future, but with the establishment of a functional alternative system and political commitment patient triage and dispatch processes will be much more effective outside of emergency rooms. Furthermore, paramedics adhere to SOPs and thus ensure standardised treatment. Telemedical physicians are well trained in respective SOPs and can contact specialists of various medical specialties on call at our university hospital 24/7.

Additionally, the law requires a doctor to personally determine death in Germany. During our study - although the out-of-hours service was provided by healthcare professionals such as a paramedics - a doctor was on-call voluntarily in the background to ensure the necessary presence of a physician in case of a post-mortem examination. This helped to make the process legally sound while improving efficiency and relieving emergency care resources.

Limitations to this study arise through the inability to properly analyse technical parameters. Nothing is known about the parameters that guided the decision-making process of healthcare professionals on site. A further analysis, including the weighting of different parameters (e.g. ECG, oxygen saturation, audio, video) would be beneficial for a more comprehensive understanding of which cases would benefit from telemedical support and the underlying reasons for that. Furthermore, it is currently unclear how many patients and missions can be handled by one single telemedicine doctor on call. This would be important in order to calculate how many telemedicine doctors are needed per 100,000 inhabitants for expansion to other districts.

As the dataset shown in Table 1 contains only basic data further research is needed to answer in-depth questions of health services research.

Ensuring a secure mobile phone connection at all times remains a significant challenge, particularly in rural Germany [17]. This is necessary for vital data transmission and personal doctor-patient consultations, which are essential for the trust and acceptance of this novel form of care. Without it, the expansion of telemedicine in rural settings will be limited, and the current system with doctors on site will remain the primary option.

## Conclusion

In conclusion, the system described in our study presents a feasible and promising alternative to the existing GP on-call system. In the context of the growing shortage of doctors, an on-call system not relying on doctors on site would be an effective means of conserving precious medical resources. The expertise of medical professionals would not be lost but rather be provided in a sufficient and targeted manner via telemedical support. Our findings indicate that telemedicine is a beneficial approach in the majority of cases, in particular for patients presenting with infectious/parasitic diseases, cardiocirculatory problems, or musculoseletal disorders.

## Data Availability

The datasets used and/or analyzed during the current study are available from the corresponding author on reasonable request.

## Abbreviations

ESF: European Social Fund
GP: General practitioner
GSM: Global System for Mobile Communications
JUH: Johanniter Unfall-Hilfe e.V
KOL: Klinikum Oldenburg AöR
KVN: Kassenärztliche Vereinigung Niedersachsen
SOP: Standard Operating Procedure
